# Electrophysiological and fundoscopic detection of intracranial hypertension in craniosynostosis

**DOI:** 10.1038/s41433-021-01839-w

**Published:** 2022-01-01

**Authors:** Sohaib R. Rufai, Oliver R. Marmoy, Dorothy A. Thompson, Lara S. van de Lande, R. William Breakey, Catey Bunce, Vasiliki Panteli, Kemmy Schwiebert, Shafquet Mohamed, Frank A. Proudlock, Irene Gottlob, David J. Dunaway, Richard Hayward, Richard Bowman, Noor ul Owase Jeelani

**Affiliations:** 1 Clinical and Academic Department of Ophthalmology, Great Ormond Street Hospital for Children NHS Foundation Trust, London, WC1N 3JH UK; 2 UCL Great Ormond Street Institute of Child Health, London, WC1N 1EH UK; 3 The University of Leicester Ulverscroft Eye Unit, Leicester Royal Infirmary, Robert Kilpatrick Clinical Sciences Building, PO Box 65, Leicester, LE2 7LX UK; 4 Craniofacial Unit, Great Ormond Street Hospital for Children NHS Foundation Trust, London, WC1N 3JH UK; 5 Clinical Trials Unit, The Royal Marsden NHS Foundation Trust, London, SW3 6JJ UK

**Keywords:** Predictive markers, Optic nerve diseases, Neurological disorders

## Abstract

**Aims:**

To assess the diagnostic accuracy of fundoscopy and visual evoked potentials (VEPs) in detecting intracranial hypertension (IH) in patients with craniosynostosis undergoing spring-assisted posterior vault expansion (sPVE).

**Methods:**

Children with craniosynostosis undergoing sPVE and 48-hour intracranial pressure (ICP) monitoring were included in this single-centre, retrospective, diagnostic accuracy study. Data for ICP, fundoscopy and VEPs were analysed. Primary outcome measures were papilloedema on fundoscopy, VEP assessments and IH, defined as mean ICP > 20 mmHg. Diagnostic indices were calculated for fundoscopy and VEPs against IH. Secondary outcome measures included final visual outcomes.

**Results:**

Fundoscopic examinations were available for 35 children and isolated VEPs for 30 children, 22 of whom had at least three serial VEPs. Sensitivity was 32.1% for fundoscopy (95% confidence intervals [CI]: 15.9–52.4) and 58.3% for isolated VEPs (95% CI 36.6–77.9). Specificity for IH was 100% for fundoscopy (95% CI: 59.0–100) and 83.3% for isolated VEPs (95% CI: 35.9–99.6). Where longitudinal deterioration was suspected from some prVEPs but not corroborated by all, sensitivity increased to 70.6% (95% CI: 44.0–89.7), while specificity decreased to 60% (95% CI: 14.7–94.7). Where longitudinal deterioration was clinically significant, sensitivity decreased to 47.1% (23.0–72.2) and specificity increased to 100% (47.8–100). Median final BCVA was 0.24 logMAR (*n* = 36). UK driving standard BCVA was achieved by 26 patients (72.2%), defined as ≥0.30 logMAR in the better eye.

**Conclusion:**

Papilloedema present on fundoscopy reliably indicated IH, but its absence did not exclude IH. VEP testing boosted sensitivity at the expense of specificity, depending on method of analysis.

## Introduction

*Craniosynostosis* is characterised by the premature fusion of the cranial sutures. Its complex forms are commonly associated with intracranial hypertension (IH) that, if left untreated, can cause cognitive impairment, visual impairment and even death [1]. The estimated prevalence of craniosynostosis is 3.1–6.4 in 10,000 births and rising [2]. It can be sub-classified as non-syndromic (approximately one-third of cases) that affects one or multiple sutures, or syndromic (approximately two-thirds of cases), the majority of whom have multiple suture fusions often combined with extracranial anomalies [3]. IH occurs in 30–40% [4, 5] of the syndromic cases, most commonly in patients diagnosed with Apert (71%) [6], Crouzon (61%) [7] and Pfeiffer (60%) [8] syndromes. In the non-syndromic cases, IH occurs in approximately 17% [9] with a single fused suture and in 24–47% [1, 8] with multiple sutures involved.

Surgical management of IH primarily involves expansion of the skull vault, once hydrocephalus has been excluded. This reduces the risk of associated sequelae by increasing the intracranial volume. It also allows some correction of a brachycephalic skull shape. At Great Ormond Street Hospital for Children (GOSH), the procedure of choice is a spring-assisted posterior vault expansion (sPVE) [10]. It is a less invasive operation than a fronto-orbital reconstruction, providing a greater degree of vault expansion [11] and leaving the frontal region intact for any subsequent fronto-facial surgery.

Detecting IH in children is challenging. Direct intracranial measurement of intracranial pressure (ICP) is the gold standard, but this involves hospital admission and general anaesthesia as well as the risks of infection, bleeding, cerebrospinal fluid leakage and mechanical failure [5]. Non-invasive ophthalmological methods of detecting ICP employed at GOSH include fundoscopy [12] and visual evoked potentials (VEPs) [13], performed according to a surveillance protocol [14]. The patient’s visual acuity (VA), comprehensive neurosurgical evaluation and radiological findings [15] are also considered but, when concerns remain, 48-h ICP monitoring is performed [16].

The primary objective of this study was to assess the diagnostic accuracy of ophthalmological monitoring methods (fundoscopy and VEPs) in detecting IH in a cohort of children with craniosynostosis who underwent sPVE after ICP monitoring. Our secondary objective was to evaluate visual outcomes.

## Materials and methods

### Study design and participants

This was a diagnostic accuracy study of patients with craniosynostosis undergoing ICP assessment and sPVE surgery at GOSH—a quaternary paediatric referral centre in the United Kingdom. Data were collected retrospectively between 16 February 2002 and 12 March 2019, to capture all children who underwent sPVE since the technique was started at GOSH. This study is reported according to the Standards for Reporting of Diagnostic Accuracy Studies (STARD) guidelines [17]. This study adhered to the tenets of the Declaration of Helsinki. Ethical approval was obtained for this study (UK REC 15/LO/0386—Research Ethics Committee approval—Study No. 14DS25).

Inclusion criteria were as follows: (i) children diagnosed with craniosynostosis undergoing sPVE surgery; (ii) availability of 48-h ICP assessment(s); (iii) availability of fundoscopic examination and/or VEP assessment within at least 6 months of the ICP assessment. Wherever children had three serial VEP assessments, where the most recent fell within 6 months of the ICP assessment, these were also included in the diagnostic accuracy testing. Children with fundoscopic examinations and/or VEP assessments more than 6 months prior to their ICP assessment were excluded from diagnostic accuracy testing, as those exceeding this time-frame were unlikely to be reflective of true change. Adults aged 18 or over were excluded.

The following baseline characteristics were recorded: diagnosis, gender, age at first presentation at GOSH, age at first sPVE surgery, age at first bolt ICP assessment, age at final follow-up, total follow-up and final destination. Diagnoses were based on genetic testing, radiological findings and clinical assessment. Final destination was defined as further follow-up at GOSH, transfer to local hospital or discharge to community opticians.

### Primary outcome measures

The primary outcome measures were papilloedema on fundoscopy, VEP assessments and 48-h ICP assessments. Diagnostic accuracy was assessed for fundoscopy and VEP against IH (present/absent).

#### Fundoscopy

Fundoscopy was performed by experienced paediatric neuro-ophthalmologists (RB and VP). Examinations immediately prior to ICP assessment and at final visit were recorded. Optic disc examinations were graded from the clinical notes as ‘normal’, ‘swollen’, ‘mild pallor’ or ‘atrophic’. Frequency of fundoscopic examinations was as per our surveillance protocol [14]; only examinations within 6 months of bolt ICP examination were included.

#### VEP methods and analysis

Pattern reversal VEPs (prVEPs) were recorded to high-contrast black and white reversing checkerboards presented on a plasma display panel subtending a 30° field of mean luminance 82 cd/m^2^ in accordance with International Society for Clinical Electrophysiology of Vision (ISCEV) standards [18]. Check widths presented with both eyes open ranged between 200’ and 6.25’ depending on the age, ability and normality of responses. Ag-AgCl electrodes were applied with conductive paste over the occipital scalp at Oz, with a reference electrode placed at Fz and ground at Cz. Signals were filtered between 0.3 and 100 Hz with a minimum of 50 trials obtained in a minimum of two averages, unless patient cooperation limited this. All data were acquired with an Espion E^3^ system (Diagnosys LLC, Cambridge, UK).

VEPs were scheduled in accordance with our surveillance protocol [14]. Two independent reviewers (ORM and DAT), masked to the other outcome measures including fundoscopy and ICP, retrospectively analysed and graded the prVEPs in two ways. The first was an analysis of prVEPs to a single check width (50’) from the recording closest to, and within 6 months of the ICP bolt measure. This ‘isolated’ single check, single episode analysis allows comparison with previously published data and the ISCEV VEP standard [18].

In this ‘isolated’ analysis, prVEP abnormality was determined relative to the 95% laboratory reference ranges for the ISCEV large check, which in this study were a latency of 90–116 ms and an amplitude of 5–62 µV. Measured prVEPs were graded using the criteria created by Thompson et al. [13]. This published prVEP grading system for 50’ check widths was modified slightly to reclassify grade 3 as ‘abnormal amplitude’ rather than ‘reduced amplitude’, and grade 4 as ‘abnormal peak-time’ rather than ‘delayed peak-time’. This allowed us to account for prVEPs which were atypically large (i.e., ‘giant VEPs’, *n* = 1) exceeding the reference limit (>62 µV) and those of atypically early peak times (*n* = 1) earlier than reference limits (>90 ms), which suggests a paramacular dominance of the VEP with reduced central field sensitivity (Table 1). For the sensitivity/specificity analysis, VEP grades 1 or 2 were considered normal whilst grades 3–6 were considered abnormal as their values fell outside the laboratory reference range for amplitude and/or peak-time.

**Table 1:** VEP grading criterion modified from Thompson et al. (2006) [13].

VEP grade	Modified VEP grading criteria
Grade 1	Normal VEP (amplitude and latency within normal limits)
Grade 2	Normal amplitude and latency, but broadened waveform
Grade 3	Abnormal amplitude (low amplitude or atypically ‘giant’) with normal latency
Grade 4	Normal amplitude with abnormal latency (prolonged or atypically early latency)
Grade 5	Abnormal amplitude and latency
Grade 6	No VEP response

*VEP* visual evoked potentials.

The second analysis undertaken was a longitudinal inspection of prVEPs to a range of check widths [200’, 100’, 50’, 25’, 12.5’ and 6.25’] recorded in three successive appointments prior to the ICP assessment, the closest within 6 months of the ICP bolt. The longitudinal stability of prVEPs was graded as follows: 0 = stable, 1 = equivocal, 2 = deterioration and –1 = improvement. A clinically significant deterioration was regarded if a cumulative score over visits was ≥2, a subtle/suspicious deterioration if the cumulative score was ≥1.

The consideration of ‘stability’ for patients with craniosynostosis included prVEP data produced to all check widths. Changes in measured amplitude, peak-time and waveform morphology across visits were noted. In cases where prVEPs were degraded (i.e., grades 5/6), stability was determined using pattern onset or flash VEPs when recorded. ‘Equivocal’ prVEPs are those suspicious of deterioration to some check widths, but not corroborated across all checks, and include a qualitative assessment of test compliance within the quantitative change. It is summarised broadly as follows:

- A P100 amplitude change of ≥30% in no more than 1–2 check widths
- A latency change of >5 ms of P100 to 1–2 check widths (typically small checks—12.5’/6.25’)
- A change of >50% in amplitude or latency across ≥2 check widths, but with sub-optimal prVEP test compliance

#### ICP assessment

Patients were admitted for gold standard, 48-hr ICP assessment as inpatients following ophthalmological monitoring. The RAUMEDIC ICP bolt (RAUMEDIC AG, Helmbrechts, Germany) was used. ICP was assessed over 48-hr and reported by a consultant specialising in ICP sleep studies and/or experienced consultant craniofacial surgeons (NuOJ and DJD), all of whom had access to the ophthalmological monitoring results as per the usual GOSH clinical policy. We considered mean ICP values between 10 and 20 mmHg as within the normal range in children with craniosynostosis [16, 19]. Mean ICP values ≥20 mmHg were considered raised and classified as IH.

### Secondary outcome measures

Our secondary outcome measures were final BCVA and the proportion of children achieving UK driving standard BCVA (≤0.30 logMAR) [20]. Final BCVA was measured at final visit using Thomson LogMAR test charts (Thomson Software Solutions, Hatfield, UK) wherever possible; if not possible, then BCVA was tested using forced preferential looking using Keeler Acuity Cards (Keeler Ltd, Windsor, UK), wherever possible.

### Additional outcome measures

Prevalence of amblyogenic risk factors was reported. Amblyogenic risk factors were defined as per the American Association of Pediatric Ophthalmology and Strabismus Guidelines [21].

### Statistical analysis

Sensitivity, specificity, positive predictive value, negative predictive value and accuracy were calculated for fundoscopy and VEPs against IH (present/absent) based on initial ICP assessment. Accuracy is defined as the proportion of true test results (either true positives or true negatives) amongst all evaluated cases. Calculation of sample size was not applicable given the retrospective nature of this study, rather all eligible children were included to maximise power and avoid selection bias.

## Results

### Baseline characteristics

Table 2 displays the baseline demographics of this cohort. No patients were lost to follow-up. At final visit, 13 patients (31.1%) had their care transferred from GOSH to local hospitals, while 4 patients (10.8%) were discharged into community optician care.

**Table 2:** Baseline demographics.

Baseline characteristics	Number of patients	Median (IQR)	Range
*Diagnosis*			
Syndromic			
Crouzon	8	–	–
Apert	5		
Pfeiffer	4		
ERF	1		
Muenke	1		
Smith–Lemli–Opitz	1		
Williams	1		
Total	22		
Non-syndromic			
Multisuture	15		
*Gender*			
Male	24	–	–
Female	13		
*Age at first presentation (months)*	37	3.8 (1.5, 21.1)	0.1–59.1
*Age at first sPVE surgery (months)*	37	35.5 (9.6, 58.6)	2.1–85.2
*Age at first pre-ICP clinical exam (months)*	37	52.8 (29.8, 71.0)	4.2–152.7
*Age at first ICP assessment (months)*	37	55.7 (31.5, 73.3)	4.4–154.1
*Age at final clinical exam (months)*	36	88.7 (66.6, 122.4)	20.3–185.9
*Total follow-up (months)*	36	80.9 (53.7, 104.7)	18.5–156.2

*IQR* interquartile range, *sPVE* spring-assisted posterior vault expansion.

### Diagnostic accuracy testing

**Fig. 1:**
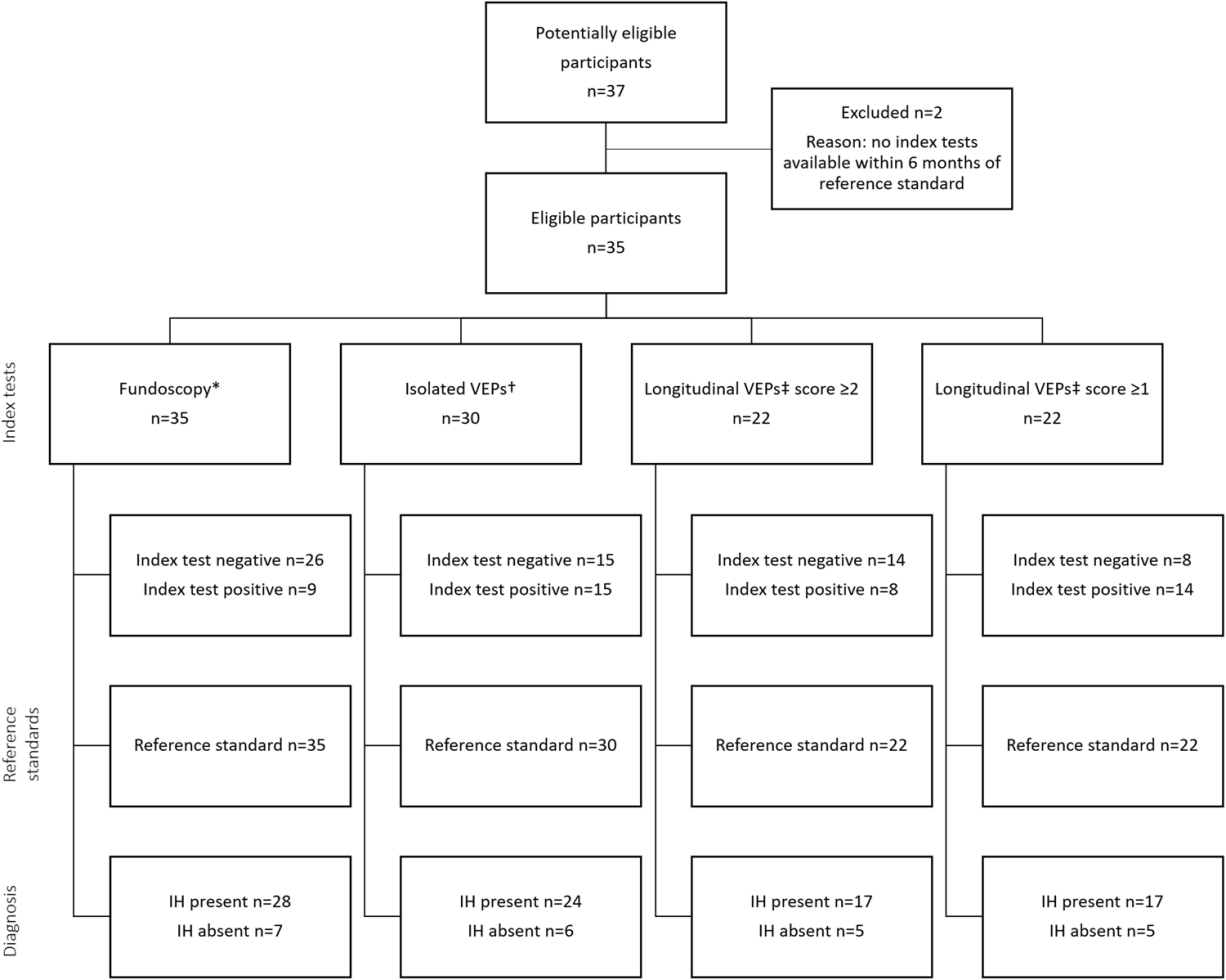
STARD patient flowchart. *Fundoscopy performed within 6 months of ICP assessments. ^†^Isolated VEPs recorded at visit immediately prior to and within 6 months of ICP assessment. ^‡^Longitudinal VEPs recorded as per isolated, plus two preceding visits; thresholds for deterioration defined as cumulative score of ≥2 and ≥1. IH intracranial hypertension, STARD standards for reporting diagnostic accuracy studies, VEP visual evoked potentials.

Prevalence of IH was 80.0% (*n* = 28; 95% confidence interval [CI]: 63.1–91.6%) based on ICP assessments available in 35 patients included in diagnostic accuracy testing. Figure 1 displays the STARD patient flowchart for our diagnostic accuracy testing.

**Fig. 2:**
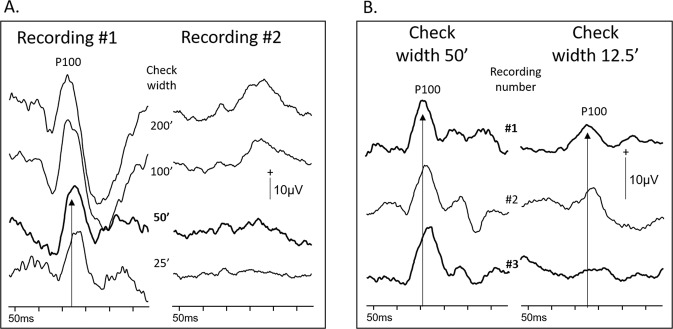
prVEP waveforms from two patients demonstrating deterioration over time. **A** Isolated analysis reveals a marked amplitude reduction to prVEPs produced to a range of different check widths, including abnormal prVEPs to 50’ checks (in bold) between recording #1 and #2. At baseline, recording #1 the 50’ check P100 latency is equivocal, borderline for age, but amplitudes are normal. **B** Longitudinal analysis displays group averaged prVEPs recorded to the ISCEV large (50’) and small (12.5’) checks on three consecutive visits. Deterioration is evident as an increasing delay in P100 latency to small checks between recordings #1 and #2: a delay of +4 ms to 50’, (+9 ms for 25’ not shown) and +12 ms for 12.5’ check widths. Further deterioration between recordings #2 and #3 is noted as reduction in P100 amplitude to the small checks 12.5’ (and 6.25’ not shown). prVEP pattern reversal visual evoked potential, ISCEV International Society for Clinical Electrophysiology of Vision.

Fundoscopic examinations during appointments within the prior 6 months of first ICP assessments were available in 35 patients. Median time between pre-ICP fundoscopy and first ICP assessment was 1.7 months (IQR: 2.1). Baseline VEP data were available for 35 patients; 30 recordings were within 6 months of the ICP measurement and three serial VEP studies available for 22 patients. Median time between most recent VEP and first ICP assessment was 1.5 months (IQR: 2.3). Table 3 displays 2 × 2 contingency tables and diagnostic accuracy testing of fundoscopy and VEPs against IH. Figure 2 displays prVEP waveforms from two sample patients demonstrating deterioration over time. Supplementary Table 1 (Supplementary Material) displays raw VEP data.

**Table 3:** 2 × 2 contingency tables.

2 × 2 tables	IH		Sensitivity (95% CI)	Specificity (95% CI)	PPV (95% CI)	NPV (95% CI)	Diagnostic accuracy (95% CI)
	Present	Absent					
Fundoscopy^a^ (*n* = 35)			32.1% (15.9–52.4)	100% (59.0–100)	100% (70.1–100)	26.9% (22.2–32.2)	45.7% (28.8–63.4)
PE	9	0					
Non-PE	19	7					
Isolated VEPs^b^ (*n* = 30)			58.3% (36.6–77.9)	83.3% (35.9–99.6)	93.3% (69.4–98.9)	33.3% (21.6–47.5)	63.3% (43.9–80.1)
Abnormal	14	1					
Normal	10	5					
Longitudinal VEPs^c^ score ≥ 2 (*n* = 22)			47.1% (23.0–72.2)	100% (47.8–100)	100% (67.6–100)	35.7% (26.2–46.5)	59.1% (36.4–79.3)
Abnormal	8	0					
Normal	9	5					
Longitudinal VEPs^c^ score ≥ 1 (*n* = 22)			70.6% (44.0–89.7)	60.0% (14.7–94.7)	85.7% (66.3–94.8)	37.5% (17.7–62.6)	68.2% (45.1–86.1)
Abnormal	12	2					
Normal	5	3					

*CI* confidence interval, *IH* intracranial hypertension, *non-PE* non-papilloedematous, *PE* papilloedematous, *NPV* negative predictive value, *PPV* positive predictive value, *VEP* visual evoked potentials.

^a^Fundoscopy performed within 6 months of ICP assessments.

^b^Isolated VEPs recorded at visit immediately prior to and within 6 months of ICP assessment.

^c^Longitudinal VEPs recorded as per isolated, plus two preceding visits; thresholds defined as cumulative score of ≥2 and ≥1.

Out of the nine patients with papilloedema on fundoscopy prior to ICP assessment, all disc swelling had resolved on final examination. One of these nine patients was noted to have mild bilateral disc pallor, while another had mild unilateral disc pallor on final examination. None of these nine patients developed optic atrophy.

### Secondary outcome measures: visual outcomes

Final visual outcomes were available for 36 out of 37 children (97.3%). Final BCVA data were recorded by logMAR chart vision testing in 32 children (88.9%); 3 children (8.3%) who could not participate with chart testing had forced preferential looking, while 1 child (2.8%) had perception of light only (logMAR equivalent: 2.70) [22]. Data were unavailable for one child as they were an international patient transferred back to their home country before final ophthalmological examination and BCVA.

Median final BCVA was 0.24 logMAR (IQR: 0.51; range: –0.06 to 2.7). UK driving standard BCVA was achieved by 26 patients (72.2%), defined as ≥0.30 logMAR in the better eye [20]. Two patients (5.6%) had visual impairment and two patients (5.6%) had severe visual impairment, defined as 1.0–2.0 logMAR and ≥2.00 logMAR in the better eye, respectively [23]. Non-syndromic patients had better final BCVA (median: 0.20 logMAR; IQR: 0.36; range: –0.06 to 1.45) as compared to syndromic patients (median: 0.30 logMAR; IQR: 0.43; range: –0.04 to 2.70). This difference was statistically significant (Mann–Whitney *U* test; z-score 2.11; *p* = 0.03).

### Additional outcome measures

There was a high prevalence of amblyogenic factors in this cohort, including manifest strabismus (52.8%), astigmatism >1.5 D (34.4%) and V-pattern (25%). Supplementary Table 2 (Supplementary Material) provides a full breakdown of amblyogenic factors.

## Discussion

To the best of our knowledge, this is the largest study of patients with craniosynostosis undergoing gold standard invasive ICP monitoring with serial ophthalmological examinations to understand how these measurements change with IH. Fundoscopy demonstrated low sensitivity (32.1%; 95% CI: 15.9–52.4) and high specificity (100%; 95% CI: 59.0–100) for IH. Isolated VEPs demonstrated 58.3% sensitivity (95% CI 36.6–77.9) and 83.3% specificity (95% CI: 35.9–99.6). Where longitudinal deterioration was subtle/suspicious (cumulative score ≥1), sensitivity increased to 70.6% (95% CI 44.0–89.7), while specificity decreased to 60% (95% CI: 14.7–94.7%). Where longitudinal deterioration was clinically significant (cumulative score ≥2), sensitivity decreased to 47.1% (95% CI 23.0–72.2), while specificity increased to 100%. Papilloedema resolved in all nine affected patients by final examination. Median final BCVA was 0.24 logMAR (IQR: 0.51; range: –0.06 to 2.7). On final visit, the majority of children (72.2%) achieved UK driving standard vision, defined as ≥0.30 logMAR in the better eye [20]. There was a significant prevalence of amblyogenic risk factors, as expected in this patient population [14, 24].

### Ophthalmological monitoring

This study has highlighted the role of ophthalmological monitoring to detect IH and promptly refer for sPVE surgery, albeit no method displayed 100% sensitivity. When used as part of a multidisciplinary approach, ophthalmological evaluation may prompt neurosurgical evaluation and timely surgical intervention as appropriate.

Our study found that fundoscopic observation of papilloedema reliably indicated IH, but its absence did not exclude IH. PrVEPs were more helpful in detecting IH. PrVEPs had a high sensitivity of 70.6% for detecting IH when based on the stability of three serial VEPs. It is an important consideration that isolated VEP abnormalities observed within six months of IH detection only had a moderate sensitivity (58.3%), as clinically we may observe a deterioration in VEPs that remain within normal limits; therefore, longitudinal monitoring is essential in patients at risk of IH. The higher sensitivity of longitudinal VEPs most likely reflects the wide range of check widths used for VEP testing, which includes, but extends the ISCEV standard, and use of the individual patient as their own control. Often the earliest change is noted in the prVEP produced by the smallest check widths in the previous recording of the individual’s VEPs, suggesting a range of check widths is beneficial in monitoring IH [18]. The benefits of prVEPs over other methods of ophthalmic monitoring, such as VA or fundoscopy, in young children is that their values typically reach within 10% of adult values by 6 months of age [25]. Therefore, where fundoscopy is subjective and VA matures over a longer time period requiring different techniques of assessment, the VEP can be used throughout childhood as a measurement of visual pathway stability. Regarding whether VEP reflects IH versus optic atrophy, we feel there may be a complex interplay between both; we can observe improvement of VEPs following surgical intervention, suggesting IH rather than atrophy; however, some do not improve to the baseline level after deterioration, perhaps reflecting a degree of optic atrophy. Finally, it should be noted that VEP results should be interpreted relative to locally derived VEP reference ranges, as these are dependent on the local stimulus and recording parameters of each laboratory.

### Comparison with existing literature

#### Visual evoked potentials

The VEP grading criteria by Thompson et al. [13] were modified in this study according to the experience of our laboratory since the initial publication of these criteria. We have observed that ‘giant’ (i.e., atypically large) VEP amplitudes can be associated with patients with IH. Furthermore, early peak times suggest enhancement of the paramacular pattern VEP contributions, which would suggest macular pathway dysfunction—something we also note clinically. Our finding of sensitivity 70.6% is similar to recent studies by Haredy et al. [26, 27] who conducted a retrospective study in a smaller sample of 13 children with craniosynostosis, demonstrating a sensitivity of 71.4% for detection of IH, but with 100% specificity compared to 60.0% specificity in our group of 22 patients. Using serial prVEPs in their later publication [27], eight of nine patients with invasively detected IH had abnormal VEPs. Our study used a range of check widths and serial prVEP recordings and, with the findings of Haredy et al. [27], support the improved sensitivity afforded by the longitudinal change to small check widths compared to a prVEP to a single check width alone. PrVEPs produced by small check widths are altered early in optic nerve dysfunction in other diseases [25]. Our findings suggested that a range of check widths for serial prVEP monitoring of patients at risk of IH is particularly useful. These study findings corroborate the benefits of prVEPs to assess the functional integrity of the macular pathway, which may be affected in early IH, rather than depending upon the subjective observation of frank papilloedema, which is a specific manifestation of IH. The VEP is a signal detected from the cortex after traversing the entirety of the macular pathway. As such, it is susceptible to changes in ICP; moreover, prVEPs to large stimuli occupy a larger field than VA measurements. The VEP can be attenuated with reduced contrast sensitivity and by field changes, whereas VA may remain insensitive as it is a high-contrast test influenced by cognitive association. Further work is needed to explore the role of VEP monitoring in other causes of paediatric IH.

We used both quantitative and qualitative interpretation of VEPs to determine stability in our cohort. In some circumstances quantitative values would indicate a deterioration but when overlaying serial waveforms to compare morphology, it became more evident that measurements could be influenced by background noise or be inconsistent across different check widths. This may explain the lower sensitivity in our series than reported by Haredy et al.

The mechanism behind the earlier change in prVEPs to smaller checks relative to larger checks or VA measurements is likely complex, but probably reflects some multiplicity of reduced contrast sensitivity, modified spatial tuning function due to subtle changes in axonal physiology in the optic nerve that are not reflected in measurements of recognition VA, which is a high-contrast measurement influenced by higher cortical processing. These findings corroborate the benefits of prVEPs in multidisciplinary assessments of craniofacial children as they assess the entirety of the macular pathway that may become dysfunctional or modified from changes in early ICH, rather than the observation of papillodema alone that is manifestation of frank ICH and subjective in nature.

#### Fundoscopy

Tuite et al. [12] conducted a large study of 122 children with craniosynostosis who underwent fundoscopy and ICP monitoring. They found excellent specificity (98%) for papilloedema on fundoscopy and raised ICP. Sensitivity of fundoscopy was age dependent: in children over 8 years old, sensitivity was 100%, whereas in children under 8 years old, it was only 22%. These were similar to our findings of 32.1% sensitivity and 100% specificity. Another study by Judy et al. [28] found sensitivity 17% and specificity 100% for papilloedema on fundoscopy and IH, albeit only 4 of 45 patients (8.9%) had papilloedema on fundoscopy. Tuite et al. [12] and Judy et al. [28] defined IH as ≥15 mmHg, whereas our unit now defines this as ≥20 mmHg. If we had adopted the former definition of IH in our study, only one child would have been reclassified from normal to raised ICP, but interestingly they had normal fundoscopy in all clinical visits and good final BCVA meeting UK driving standards.

### Strengths and limitations

Whilst this is perhaps the largest report of ophthalmic monitoring findings in sPVE patients with craniosynostosis undergoing invasive ICP monitoring, the sample size relative to the prevalence of these disorders still needs exploring; this may not be achieved without prospective study against patients with ICP monitoring, as our sample was possibly biased to perform ICP monitoring in those under clinical suspicion or high risk. However, a substantial proportion (20%) of ICP assessments was deemed as normal. More detailed grading of optic disc swelling, such as that offered by the Modified Frisén Scale [29], may have provided opportunity for more detailed description of disc appearance, but this was not possible due to the retrospective nature of this study.

VEP analysis and grading were performed by masked reviewers (ORM and DAT) to avoid experimenter bias. Perhaps one of the main limitations highlighted by this study was that VEPs may be more sensitive where clinicians are suspicious but uncertain of deterioration, for example, due to poor test compliance. VEPs with a cumulative score of 1 had a higher sensitivity than those with definitive deterioration (i.e., score of 2 or above). This is a clinically challenging circumstance, to identify where VEPs have deteriorated, but at the expense of higher sensitivity the specificity reduces also. The complementarity of multidisciplinary assessment has value here. Future work may hope to address this knowledge gap to identify coefficients of variability for paediatric VEP data through maturation to inform future analyses.

Different vision testing methods were used in different children, which may provide diverse estimates of true VA, albeit the majority were able to perform logMAR chart testing at final visit. Refractive data were unavailable for five patients, as these refractions were performed by local optometrists. Optical coherence tomography (OCT) was not possible in young infants in this cohort. However, our unit has recently adopted handheld OCT imaging for young infants with craniosynostosis, using a recently published image acquisition protocol [30], as well as fundus photography and B-scan ultrasound wherever possible.

### Conclusion

This study demonstrated the role of fundoscopy and VEPs in a cohort of children with craniosynostosis undergoing sPVE and ICP monitoring. Fundoscopic observation of papilloedema reliably indicated IH, but its absence did not exclude IH. VEPs demonstrated higher sensitivity in this study, but at the expense of specificity depending on method of analysis. Final visual outcomes were generally good with the majority of children achieving UK driving standard vision, but visual morbidity and amblyogenic factors remain substantial and further work is required to optimise clinical decision making and management of craniosynostosis.

### Summary

#### What was known before

- Craniosynostosis is associated with intracranial hypertension, which can manifest clinically as papilloedema.
- There is a high prevalence of abnormal pattern reversal visual evoked potentials in children with craniosynostosis.

#### What this study adds

- Papilloedema present on fundoscopy reliably indicates intracranial hypertension in young children with craniosynostosis, but its absence does not exclude it.
- Monitoring of visual evoked potentials can be valuable in detecting intracranial hypertension in children with craniosynostosis. Longitudinal interpretation can boost sensitivity for detecting intracranial hypertension in craniosynostosis, at the expense of specificity.

## Supplementary information

Supplementary Material
