# Supplementary material for: Electrophysiological and fundoscopic detection of intracranial hypertension in craniosynostosis
Source: Eye (Lond). 2022 Jan 1;37(1):139–45. doi: 10.1038/s41433-021-01839-w (PMC9829653; doi:10.1038/s41433-021-01839-w)
Supplement: Supplementary file 1 — Supplementary Material [file 41433_2021_1839_MOESM1_ESM.pdf]

Supplementary Material

Participant code	Diagnosis	Age at VEP (months)	Time before bolt (days)	VEP grade 50' (pre-ICP)	Longitudinal Score	200' amp. (µV)	200' PT (ms)	100' amp. (µV)	100' PT (ms)	50' amp. (µV)	50' PT (ms)	50' breadth (ms)	25' amp. (µV)	25' PT (ms)	12.5' amp. (µV)	12.5' PT (ms)	6.25' amp. (µV)	6.25' PT (ms)	Time between ICP + VEP (days)	Cumulative longitudinal score	Isolated VEP score (pre-ICP)	ICP result
**Patients with longitudinal datasets**																						
		108	685.00	1	0	NR	NR	23	104	23	104	74	18	115	13	125	3	122				
PVE1	Bicoronal synostosis	133	496.00	1	1	NR	NR	NR	NR	21	106	79	18	108	10	125	3	144				
		136	377.00	1	2	NR	NR	20	112	19	110	80	25	117	8 (3)	99 (137)	2	106				
		**147**	69.00	**4**	2	NR	NR	NR	NR	22	116	94	13	117	3	149	ND	ND	69	5	Abnormal	Raised
PVE2	Crouzon syndrome	6	593.00	4	B	24	111	30	112	28	124	84	20	131	NR	NR	NR	NR				
		19	208.00	5	2	3	95(161)	4 (10)	85 (163)	4	95 (168)	42	3	95	NR	NR	NR	NR				
		**25**	32.00	**5**	2	ND	ND	2	167	0	145 - 165	118	2	145 - 174	NR	NR	NR	NR	32	4	Abnormal	Raised
		16	859.00	1	0	NR	NR	NR	NR	62	109	70	63	117	44	121	11	121				
PVE3	Crouzon syndrome	31	377.00	1	0	NR	NR	NR	NR	46	102	75	58	109	25	120	13	124				
		38	180.00	1	0	NR	NR	NR	NR	55	101	78	44	108	24	117	14	124				
		**43**	20.00	**1**	0	NR	NR	NR	NR	54	98	70	61	110	52	110	19	114	20	0	Normal	Raised
PVE4	Apert syndrome	38	488.00	4	0	2(30)	103 (207)	6(12)	105 (175)	9(14)	112 (170)	50	15	136	5	155	NR	NR				
		48	187.00	4	0	9(29)	108 (189)	4(11)	106 (159)	13	111	129	NR	NR	5	152	NR	NR				
		**54**	19.00	**4**	-1	NR	NR	9	106-145	12	116	98	14	128	14	121	ND	ND	19	-1	Abnormal	Raised
PVE5	Crouzon syndrome	26	170.00	2	B	9	120	11	118	11	122	81	11	134	6	172	NR	NR				
		**31**	21.00	**4**	2	9	113	10	115	12	131	90	8	170	NR	NR	NR	NR	21	2	Abnormal	Raised
PVE6	Apert syndrome	6	300.00	4	B	24	152	17	158	16	160	83	9	166	5	160	NR	NR				
		10	196.00	4	1	13	97	13	115	11	122	87	8	113	6	100	NR	NR				
		**15**	27.00	**3**	1	8	97	14	100	6	99	105	NR	NR	NR	NR	NR	NR	27	2	Abnormal	Raised
		18	461.00	1	0	NR	NR	6	97	12	102		13	114	10	124	6	111				
PVE7	Apert syndrome	20	321.00	2	2	NR	NR	2	99	7	107	147	9	112	6	138	NR	NR				
		26	139.00	2	-1	NR	NR	13	100	13	102	70	6	114	6	123	NR	NR				
		**30**	27.00	**1**	0	NR	NR	18	106	18	104	69	17	108	11	124	8	128	27	1	Normal	Raised
PVE8	Crouzon syndrome	102	1601.00	4	0	NR	NR	NR	NR	49	92	66	36	99	26	109	19	121				
		138	488.00	4	0	NR	NR	NR	NR	38	93	69	44	98	46	111	26	128				
		**153**	33.00	**4**	1	NR	NR	NR	NR	36	89	70	32	97	34	126	22	128	33	1	Abnormal	Raised
PVE9	Muenke syndrome	39	601.00	2	0	NR	NR	9	106	9	110	39	7	107	NR	NR	NR	NR				
		45	404.00	3	0	NR	NR	11	106	14	110	41	14	114	16	116	6	124				
		**57**	47.00	**2**	0	20	120	13	104	16	110	57	14	124	NR	NR	NR	NR	47	0	Abnormal	Raised
PVE10	Crouzon syndrome	46	284.00	1	0	NR	NR	NR	NR	53	94		44	92	39	104	23	112				
		49	215.00	3	1	NR	NR	NR	NR	80	96	102	51	97	48	108	40	113				
		**54**	48.00	**3**	1	NR	NR	NR	NR	86	93	92	77	101	106	112	73	111	48	2	Abnormal	Raised
PVE11	Bartter's syndrome	37	983.00	3	0	13	140	17	140	NR	NR	NR	NR	NR	NR	NR	NR	NR				
	Multi-suture	52	572.00	3	0	24	135	9	128	12	99	62	NR	NR	NR	NR	NR	NR				
		**61**	111.00	**3**	1	11	122	8	122	6	101	67	NR	NR	NR	NR	NR	NR	111	1	Abnormal	Raised
PVE12	Multi-suture	25	624.00	5	0	degraded but evident		NR	NR	NR	NR	NR	NR	NR	NR	NR	NR	NR				
		36	267.00	6	0	Pattern onset VEPs 400' & 200' present			NR	NR	NR	NR	NR	NR	NR	NR	NR	NR				
		**41**	141.00	**6**	2	Pattern onset VEPs not detectable			Flash VEPs stable		NR	NR	NR	NR	NR	NR	NR	NR	141	2	Abnormal	Raised
		49	1026.00	4	0	21	117	14	115	11	117	85	4(7)	98(188)	NR	NR	NR	NR				
PVE13	Pfeiffer syndrome	62	614.00	5	2	21	113	16	120	11	78 (131)	134	7	147	NR	NR	NR	NR				
		64	556.00	5	-1	12	113	17	114	11	77 (122)	85	5	90 (137)	NR	NR	NR	NR				
		**78**	153.00	**5**	0	16	108	22	113	6(5)	75 (117)	103	5	91 (130)	NR	NR	NR	NR	153	1	Abnormal	Raised
PVE14	Multi-suture	12	529.00	1	B	8	86	8	104	7	101	69	5	125	NR	NR	NR	NR				
		24	181.00	1	1	10	86	7	85	7	96	77	8	89	NR	NR	NR	NR				
		**32**	-71.00	**4**	2	7	135	9	119	9	116	69	8	82	NR	NR	NR	NR	181	3	Abnormal	Raised
PVE15	Multi-suture	66	226.00	1	0	3	129	5	105	7	99	52	6	93	7	104	ND	ND				
		72	55.00	3	1	14	143	6	159	4	111	77	NR	NR	NR	NR	NR	NR				
		**73**	13.00	**3**	0	4	146	4	105	4	110	51	NR	NR	NR	NR	NR	NR	13	1	Abnormal	Normal
PVE16	Multi-suture	82	237.00	1	0	NR	NR	NR	NR	13	104	67	12	115	9	117	3	119				
		87	85.00	1	0	NR	NR	NR	NR	15	104	61	12	118	12	115	6	118				
		**89**	27.00	**1**	1	NR	NR	NR	NR	19	104	54	21	116	7	117	9	180	27	1	Normal	Normal
PVE17	Multi-suture	46	635.00	1	0	NR	NR	NR	NR	22	99	90	26	105	27	117	33	130				
		55	370.00	1	0	NR	NR	NR	NR	28	102	71	32	104	28	117	33	127				
		**67**	13.00	**1**	-1	NR	NR	NR	NR	44	100	85	41	103	46	115	26	129	13	-1	Normal	Normal
												]										
PVE18	Multi-suture	42	580.00	1	0	NR	NR	NR	NR	34	96	44	43	101	35	113	NR	NR				
		47	405.00	1	0	NR	NR	NR	NR	44	93	58	44	102	31	114	11	113				
		**57**	105.00	**1**	1	NR	NR	NR	NR	16	93	51	12	98	14	104	8	114	105	1	Normal	Normal
PVE20	ERF mutation	36	657.00	4	B	NR	NR	NR	NR	25	119	67	19	111	16	114	14	127				
	Multi-suture	44	403.00	1	0	NR	NR	NR	NR	19	99	60	20	114	20	113	19	121				
		**56**	46.00	**1**	0	NR	NR	NR	NR	29	108	57	28	108	22	116	26	123	46	0	Normal	Raised
PVE25	Apert syndrome	60	678.00	2	0	14	116	9	87	16	102	119	13	80	NR	NR	NR	NR				
		72	307.00	2	0	NR	NR	13	99	10	110	98	9	110	12	105	NR	NR				
		**81**	40.00	**2**	-1	NR	NR	NR	NR	24	109	97	34	114	9	99	8	103	40	-1	Normal	normal
PVE27	Multi-suture	19	727.00	1	0	NR	NR	14	97	14	101	79	19	110	18	113	16	120				
		32	309.00	1	0	NR	NR	NR	NR	17	99	72	19	106	32	111	14	115				
		**41**	47.00	**1**	0	NR	NR	NR	NR	20	98	63	19	104	17	109	20	115	47	0	Normal	Raised
PVE23	Pfeiffer syndrome	38	664.00	**1**	0	NR	NR	40	101	25	109	76	21	121	16	119	11	116				
		48	356.00	**2**	2	NR	NR	22	96	18	110	102	14	119	4	133	5	109(153)				
		**54**	181.00	**1**	0	NR	NR	19	95	13	111	78	14	119	11	142	NR	NR	181	2	Normal	Raised
**Patients with isolated datasets only (i.e only one VEP prior to ICP bolt)**																						
PVE21	Multi-suture	**115**	45.00	**1**	N/A	NR	NR	NR	NR	25	106	54	29	111	23	117	4	123	45	N/A	Normal	Raised
PVE22	Crouzon syndrome	**136**	54.00	**1**	N/A	NR	NR	NR	NR	28	101	67	26	105	21	112	13	109	54	N/A	Normal	Raised
PVE24	Multi-suture	**89**	35.00	**1**	N/A	NR	NR	NR	NR	44	107	77	24	105	34	114	10	108	35	N/A	Normal	Raised
PVE26	Crouzon syndrome	**9**	3.00	4	N/A	14	133	5	140	8	136	51	3	123	NR	NR	NR	NR	3	N/A	Abnormal	Raised
PVE19	Multi-suture	**34**	34.00	**1**	N/A	NR	NR	NR	NR	37	105	87	61	112	44	120	29	126	34	N/A	Normal	Raised
PVE28	Multi-suture	**25**	60.00	**1**	N/A	NR	NR	34	102	11	105	58	21	109	4	120	NR	NR	60	N/A	Normal	Raised
PVE29	Multi-suture	**5**	148.00	**4**	N/A	NR	NR	NR	NR	32	121	88	49	125	20	131	ND	ND	148	N/A	Abnormal	Raised
PVE30	Multi-suture	**17**	-9.00	**1**	N/A	NR	NR	NR	NR	39	104	68	33	117	29	141	14	163	-9	N/A	Normal	Normal
**REFERENCE RANGES**		**For patients 6-12 months old**			1st centile	8	83	9	88	4	95		7	103	7	113	5	119				
					5th centile	9	84	9	89	6	95		7	103	7	113	5	119				
					Median	44	95	53	98	62	104	70-80ms	54	116	43	126	14	133				
					95th centile	46	105	60	110	64	117		60	128	49	156	26	148				
					99th centile	52	106	62	111	66	117		61	128	50	153	26	148				
		**For patients >12 months**			1st centile	4	77	4	89	8	90		7	91	8	98	7.5	98				
					5th centile	5	82	6	90	9.5	92		8	92	8	98	7.5	101				
					Median	24	99	25	99	27	99	70-80ms	25	106	30	110	22	116				
					95th centile	48	130	61	109	50	113		50	115	66	124	59	135				
					99th centile	48	133	63	110	52	116		51	116	72	126	60	136				

eTable 1: Raw VEP data

eTable 1 tabulates raw VEP data according to participant code and respective diagnosis. The VEP grade (50’) is illustrated in accordance with the VEP grading criteria, the grade closest to the ICP bolt date (bold text) was used for analysis. The longitudinal score is the score provided to each VEP recording, B = baseline test (i.e. no prior tests for comparison), 0 = stable, 1 = equivocal, 2 = deterioration and -1 = improvement. In some circumstances the data from four VEP studies were included into the table, in particular where the first of the three serial measurements showed a deterioration. The subsequent numeric section provides amplitudes (µV), P100 peak-time (ms) performed to a range of check widths (200’-6.25’) and breadth (ms) of the prVEP waveforms to 50’ checks. NR = not recorded, ND = not detectable. Those figures with two figures (i.e. 100 (130)) indicate a double-peaked or bifid waveform, whereby each peak was measured respectively.

Amblyogenic factor	Number of patients	Percentage (%)
Anisometropia > 1.5 D*	3	9.4
Hypermetropia > 3.5 D*	5	15.6
Myopia magnitude > 3.0 D*	3	9.4
Astigmatism > 1.5 D*	11	34.4
Media opacities	1	2.8
Ptosis†	0	0.0
Manifest strabismus	19	52.8
V-pattern	9	25.0

eTable 2: Prevalence of amblyogenic factors

*Refraction data only available for 32 patients, all other data available for 36 patients;
†Ptosis defined as less than or equal to 1 mm margin reflex distance. D = dioptres.
